# How can dementia and disability be prevented in older adults: where are we today and where are we going?

**DOI:** 10.1111/joim.13227

**Published:** 2021-01-10

**Authors:** I. Lisko, J. Kulmala, M. Annetorp, T. Ngandu, F. Mangialasche, M. Kivipelto

**Affiliations:** ^1^ From the Division of Clinical Geriatrics Center for Alzheimer Research Department of Neurobiology, Care Sciences and Society Karolinska Institutet Stockholm Sweden; ^2^ Faculty of Sport and Health Sciences and Gerontology Research Center University of Jyväskylä Jyväskylä Finland; ^3^ Public Health Promotion Unit Finnish Institute for Health and Welfare Helsinki Finland; ^4^ School of Health Care and Social Work Seinäjoki University of Applied Sciences Seinäjoki Finland; ^5^ Karolinska University Hospital, Theme Aging Stockholm Sweden; ^6^ Aging Research Center Department of Neurobiology, Care Sciences and Society Karolinska Institutet and Stockholm University Stockholm Sweden; ^7^ Institute of Public Health and Clinical Nutrition University of Eastern Finland Helsinki Finland; ^8^ Ageing and Epidemiology (AGE) Research Unit School of Public Health Imperial College London London UK

**Keywords:** ageing, cognitive impairment, dementia, muscle physiology, prevention

## Abstract

Ageing of the population, together with population growth, has brought along an ample increase in the number of older individuals living with dementia and disabilities. Dementia is the main cause of disability in old age, and promoting healthy brain ageing is considered as a key element in diminishing the burden of age‐related disabilities. The World Health Organization recently launched the first risk reduction guidelines for cognitive impairment and dementia. According to recent estimates, approximately 40% of dementia cases worldwide could be attributable to 12 modifiable risk factors: low education; midlife hypertension and obesity; diabetes, smoking, excessive alcohol use, physical inactivity, depression, low social contact, hearing loss, traumatic brain injury and air pollution indicating clear prevention potential. Dementia and physical disability are closely linked with shared risk factors and possible shared underlying mechanisms supporting the possibility of integrated preventive interventions. FINGER trial was the first large randomized controlled trial indicating that multidomain lifestyle‐based intervention can prevent cognitive and functional decline amongst at‐risk older adults from the general population. Within the World‐Wide FINGERS network, the multidomain FINGER concept is now tested and adapted worldwide proving evidence and tools for effective and easily implementable preventive strategies. Close collaboration between researchers, policymakers and healthcare practitioners, involvement of older adults and utilization of new technologies to support self‐management is needed to facilitate the implementation of the research findings. In this scoping review, we present the current scientific evidence in the field of dementia and disability prevention and discuss future directions in the field.

## Graphical abstract

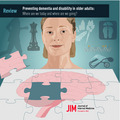

## Introduction

Extending the length of human life has been a great achievement of modern medicine. Advances in the prevention and treatment of diseases, along with societal changes, have yielded an increase in life expectancy of approximately 10 to 20 years in different regions of the world since the 1950s [1, 2]. However, population ageing and growth have led to a vast increase in the number of older individuals living with physical disability, which refers to difficulties in daily activities. In 2010, altogether 101 million older adults worldwide were dependent on others, referring to severe disability, and these numbers are projected to nearly triple by rising to 277 million in 2050 [3].

The main cause for disability amongst older adults is cognitive decline and dementia [3]. Currently, the number of individuals living with dementia is estimated to be around 50 million and the number is projected to increase to 150 million by 2050 [4]. Still in the early 1990s, high age and genetic risk factors were the only established risk factors for dementia creating a fatalistic view and giving no clear opportunities for prevention. However, during the past decades, evidence has been accumulating, indicating that several modifiable lifestyle‐related and vascular factors throughout the lifespan have a significant role for the risk of cognitive impairment and dementia [5, 6]. According to recent estimates, approximately 40% of dementia cases worldwide could be attributable to 12 modifiable risk factors: low education; midlife hypertension and obesity; diabetes, smoking, excessive alcohol use, physical inactivity, depression, low social contact, hearing loss, traumatic brain injury and air pollution, [6] indicating clear prevention potential. However, these are not risk factors only for dementia and Alzheimer’s disease (AD) but also for physical disability giving rationale for the concept of integrated interventions for these interrelated ageing‐related conditions.

Preventive measures targeted on dementia and disability are of utmost importance in halting the alarming trends projected for the increase in individuals affected by these conditions. However, the different nature between dementia and disability prevention should be recognized. Cognitive disorders and disabilities are common amongst the oldest old (often defined as 85 years and older), which is the fastest‐growing population group in the developed countries [7, 8, 9, 10]. Yet, disability is not regarded as a disease or a syndrome but rather part of the human condition [11] that majority of individuals face in old age close to death, whereas dementia is a syndrome, which can be caused by several diseases. It has been estimated that postponing dementia onset by 5 years would reduce dementia prevalence by 50% [12].

In this scoping review, we focus on epidemiological evidence and provide an overview on the current state of dementia and (physical) disability prevention and risk reduction and the future directions in the field.

## Disentangling the concepts of dementia and disability

AD is the most common form of dementia, and it accounts for about two thirds of dementia cases [13]. However, increasing evidence from neuroimaging and neuropathological studies indicates that mixed aetiologies (constituting both neurodegenerative and vascular features) serve often as underlying causes for dementia. Particularly amongst the oldest old age groups, the prevalence of mixed dementia is high and it is suggested to be the most common form [14, 15]. AD pathology and macroscopic infarctions are common also in older individuals without cognitive impairment, and the associations between neuropathology and cognition are not entirely clear [16, 17]. Most of the research on risk factors and prevention of dementia is focusing on the late‐life cognitive impairment, all‐cause dementia or AD, and there is considerably less evidence regarding other dementing diseases.

In recent years, new diagnostic criteria for AD have been proposed in order to formalize the different stages of the disease [18, 19]. Usually, AD is characterized by a long preclinical phase presenting no cognitive symptoms, followed by mild cognitive problems that can progress to overt dementia – the final and most severe stage of AD. New diagnostic frameworks integrate new advances in knowledge of the biological and clinical features of AD, with the aim to facilitate an earlier and more accurate diagnosis for AD, compared with preceding frameworks. Also regarding vascular cognitive impairment, new guidelines are currently under development in order to standardize the diagnostic classification of the aetiologically and clinically heterogeneous spectrum of cognitive impairment due to cerebrovascular disease. This progress in guidelines is reflected in the latest edition of the Diagnostic and Statistical Manual of Mental Disorders, Fifth Edition (DSM‐5) [20], where the term dementia is replaced by major neurocognitive disorder. Moreover, in the guidelines the syndromes of mild and major neurocognitive disorder recognize cognitive impairment as a spectrum.

Disability is a broad concept holding various definitions. The World Health Organization (WHO) defines disability through body functions and structures, activities and participation, and environmental factors; disability is an umbrella term for impairments, activity limitations and participation restrictions [21]. Yet, in studies the definition of disability is often based on the disablement process, in which the main pathway starts from pathology, and leads through impairments and functional limitation to disability [22]. The pathway is affected by both intra‐individual (e.g. lifestyle and behaviour changes) and extra‐individual factors (e.g. medical care and rehabilitation) and by different risk factors. Specifically, ‘Disability is defined as difficulty in doing activities in any domain of life (from hygiene to hobbies, errands to sleep) due to a health or physical problem [22]’.

Mobility is a critical characteristic of independent functioning. Mobility disability, such as the inability to walk 400 metres or climb stairs independently, is an early event in the disablement process, preceding and predicting more severe forms of disability [23]. Thus, mobility disability provides a critical target for prevention [24]. The more severe forms of disability include activities of daily living (ADL) disability. ADL may be further divided into basic ADL, including components such as dressing and undressing independently, and instrumental ADL, including components such as cleaning and maintaining the house or managing money.

Ageing associated with a decline in physiological reserves needed to maintain homeostasis, can result in a clinically recognized state of increased vulnerability, that is frailty [25], with a risk of dramatic deterioration of physical and mental well‐being (including falls, sudden change in mobility, acute confusion). In recent years, cognitive frailty [26, 27], indicating the presence of both frailty and cognitive impairment, has gained increasing interest. Altogether, disability is linked to numerous concepts describing physical functioning. In this review, we focus on studies which have namely disability as an outcome, in addition to cognitive decline and dementia.

## Shared risk factors and biological mechanisms for dementia and disability

**Fig. 1:**
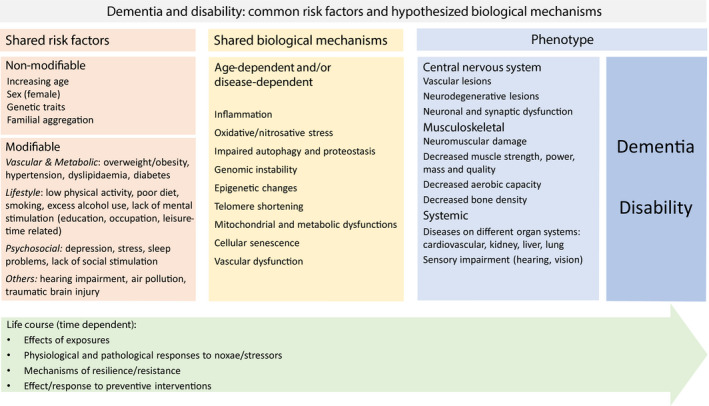
Common risk factors and hypothesized biological mechanisms for dementia and disability: modifiable factors as targets for prevention.

Several nonmodifiable and modifiable risk factors are associated with both late‐life dementia and disability [28] (Fig. 1). High age is the single most important risk factor for both. Women are more prone to the development of dementia/AD and disability [11]. The apolipoprotein‐E (*APOE*) ε4 allele is a well‐established genetic risk factor for dementia and AD [29] but it is also a risk factor for disability, indicated by a more rapid motor decline irrespective of cognitive status amongst those with *APOE* ε4 allele [30].

Cognitive and physical declines often coincide [31] but it is not clear, to which extent cognitive decline drives physical decline and vice versa [32]. Mechanisms behind dementia and disability are complex and overlapping and include both disease‐dependent and age‐dependent mechanisms (Figure 1) [28, 33, 34]. A better understanding of the ageing process can unravel the interacting pathways contributing to both cognitive and physical declines.

## Where are we today with dementia and disability prevention?

As pathology is a central concept in the disablement process, actions improving overall health and reducing morbidity are simultaneously actions that prevent disability. Furthermore, whilst morbidity results in functional decline, the association is bidirectional, with cognitive and physical decline affecting the severity and burden of diseases [35]. It is important to recognize that cognitive and physical impairment and dementia develop slowly in time, and life‐course perspective is needed to understand the potential and timing of various preventive measures.

### From observational studies to randomized controlled trials

Observational studies have provided a large amount of evidence on the possibilities of dementia and disability prevention. It has become evident that dementia and disability are multifactorial and heterogeneous conditions, driven by various genetic and environmental risk and protective factors, including vascular, psychosocial and lifestyle‐related factors. Many of these factors are potentially modifiable and provide possibilities for prevention (Figure 1).

Confirmatory evidence comes from randomized controlled trials (RCTs), which are needed to explore whether interventions targeting risk factors indicated by the observational studies can reduce the risk of dementia or cognitive decline or delay the onset of disability. Table 1 describes trials of single‐domain interventions to prevent cognitive impairment, dementia and/or physical disability. Only large (sample size of at least 500 participants) completed nonpharmacological RCTs that have cognition and/or disability as an outcome have been included in the table. Most single‐domain interventions are smaller trials, and in the following text, we will summarize the evidence from both smaller and larger trials and meta‐analyses and from observational studies.

**Table 1:** Completed large (over 500 participants) single‐domain randomized controlled trials, excluding drug trials, to prevent cognitive impairment and/or incident disability amongst older adults

Study, country	Intervention; duration	Number of participants and inclusion criteria; recruitment strategy	Outcome measures on cognition and disability	Primary outcome results	Secondary cognitive outcome results; other results/conclusions on cognitive outcomes	Secondary disability outcome results; other results/conclusions on disability outcomes
Dietary interventions						
OPAL, United States [105]	Daily supplementation of 200 mg EPA plus 500 mg DHA (omega‐3 LC PUFAs) versus olive oil placebo; 24 months	867 cognitively healthy participants aged 70–79 years; recruited from general practice records	*Primary outcome:* California Verbal Learning Test *Secondary outcomes:* Tests on memory, processing speed, reaction time and executive function	No significant differences between trial arms	No significant differences between groups in any outcome	
Physical activity interventions						
LIFE, United States [46, 48]	Moderate‐intensity intervention including walking, resistance training and flexibility exercises versus health education control; 24 months	1635 participants aged 70–89 years who were sedentary and at risk of mobility disability; recruited using various recruitment strategies	*Primary outcome:* Major mobility disability (performance‐based loss of ability to walk 400 m in 15 minutes) *Secondary outcomes:* persistent mobility disability (two consecutive major mobility disability assessments or major mobility disability followed by death); cognition measured as Digit Symbol Coding task and the revised Hopkins Verbal Learning Test *Tertiary outcomes:* global and executive cognitive function and incident MCI or dementia at 24 months	Intervention reduced incident major mobility disability (HR: 0.82, 95% CI: 0.69–0.98, *P *= 0.03)	No significant differences between groups in any cognitive outcomes; in subgroup analyses, the intervention had a beneficial effect amongst those aged ≥ 80 years and amongst those with a low level of physical activity at baseline on executive function composite scores compared with the reference group (*P *= 0.01 for interaction for both comparisons)	Intervention reduced persistent mobility disability (HR: 0.72, 95% CI: 0.57–0.91, *P *= 0.006)
Cognitive training interventions						
ACTIVE, United States [60, 61, 62, 152]	Intervention on memory training versus reasoning training versus speed of processing versus control with no contact; ten sessions of training during 5–6 weeks + four booster sessions for a subsample at months 11 and 35; 2‐year outcome and follow‐up at 5 years and 10 years	2832 participants (volunteers) aged ≥ 65 years; recruited from senior housing, community centres and hospital/clinics in 6 metropolitan areas in the United States	*Primary outcome:* daily functioning *Proximal outcomes:* memory (episodic verbal memory tasks), reasoning (identification of patterns) and speed of processing	No effects on daily functioning detected at 2 years of follow‐up; at 5 years of follow‐up, reasoning group, but not speed of processing training nor memory training, reported less difficulty in IADL than the control group (ES: 0.29, 99% CI: 0.03–0.55); at 10 years of follow‐up, each intervention group reported less difficulty with IADLs	Each intervention improved targeted cognitive ability compared with baseline, durable to 2 years (*P *< 0.001 for all); effects of interventions on the targeted cognitive ability were maintained through 5 years; cognitive training did not affect rates of incident dementia after 5 years of follow‐up; reasoning training and speed of processing training but not memory training improvement in trained cognitive ability was retained after 10 years	
Healthcare interventions						
Fletcher et al., United Kingdom [96]	Intervention comparing (1) universal versus targeted assessment and (2) subsequent management by hospital outpatient geriatric team versus primary‐care team; follow‐ups at 18 and 36 months	33 326 participants aged ≥ 75 years; individuals at long‐term care and/or terminally ill excluded; Recruitment at 106 general practices in the United Kingdom; a cluster‐randomized factorial trial	*Primary outcomes:* mortality, admissions to hospital and institution, and quality of life *Secondary outcomes*: ADL and mobility			During 36‐month follow‐up, significant improvements in mobility from the management by geriatric team versus primary‐care team (ES: –0·144, 99% CI: –0·268 to –0·020); no effect on ADL ES: –0·058 (–0·187 to 0·070); due to low ES, different forms of multidimensional assessment offered almost no differences in mobility or other patient outcomes
U‐PROFIT, The Netherlands [97]	A three‐arm intervention including 1) frailty screening (periodic) followed by best practice care versus 2) frailty screening and nurse‐led care programme consisting of a comprehensive geriatric assessment, evidence‐based care planning, care coordination and follow‐up versus 3) usual care (control); 12 months	3092 frail community‐dwelling participants aged ≥ 60 years; recruited from primary‐care networks with ~ 70 practices in Utrecht, the Netherlands; cluster randomization	*Primary outcome:* daily functioning using the Katz‐15 (6 ADLs, 8 IADLs, one mobility item)	In both intervention, arms less decline in daily functioning than amongst those in the usual care arm at 12 months; mean Katz‐15 score: screening arm, 1.87, 95% CI: 1.77–1.97; nurse‐led care arm, 1.88, 95% CI: 1.80–1.96; control group, 2.03, 95% CI: 1.92–2.13; *P *= 0.03).		Despite the statistically significant effect, the clinical relevance is uncertain because of the small differences
Stuck et al., Switzerland [98]	Intervention of in‐home visits including multidimensional geriatric assessment and quarterly follow‐up versus control (no contacts); 3‐year follow‐up	791 community‐dwelling participants aged ≥ 75 years; stratified randomized trial; stratification by risk of nursing home admission (low versus high based on 6 baseline predictors); recruitment from a health insurance list of community‐dwelling individuals aged ≥ 75 years living in 3 areas in Bern	*Primary outcome:* ADL disability (basic and instrumental) *Secondary outcomes:* cognitive functioning (MMSE score), gait and balance	At low baseline risk, participants in the intervention group had less ADL disability compared with control (OR: 0.6, 95% CI: 0.3–1.0; *P *= 0.04); at high baseline risk, no intervention effects on ADL	No intervention effects on cognitive functioning	

CI, confidence interval; DHA, docosahexaenoic acid; EPA, eicosapentaenoic acid; ES, effect size; HR, hazard ratio; IADL, instrumental activities of daily living; LC PUFA, long‐chain polyunsaturated fatty acids; MCI, mild cognitive impairment; MMSE, Mini‐Mental State Examination; OPAL, The Older People And n–3 Long‐chain polyunsaturated fatty acids Study; U‐PROFIT, Utrecht PROactive Frailty Intervention Trial (U‐PROFIT).

### Physical activity and exercise

Longitudinal observational studies have shown that physically active individuals are less likely to develop cognitive decline, all‐cause dementia, vascular dementia and Alzheimer’s disease as compared to inactive individuals [36, 37, 38, 39]. Physical activity has also been shown to prevent and slow down the disablement process amongst community‐dwelling nonfrail and moderately frail older adults [40, 41]. Based on a meta‐analysis of 16 prospective studies, physical activity was linked with a reduced risk of AD [37]. However, contradictory observations have also been made, suggesting that reverse causality may explain part of the association between physical activity and cognition [42].

When looking at single‐domain interventions, physical activity and exercise provide the strongest and most consistent evidence on the beneficial effects on cognition and physical functioning. The beneficial effects on cognitive outcomes apply to both aerobic exercise and resistance training and appear to exist regardless of cognitive status [43, 44, 45]. The Lifestyle Interventions and Independence for Elders (LIFE) study has shown that a 2‐year moderate‐intensity intervention including walking, resistance training and flexibility exercises reduced the risk of mobility disability amongst sedentary older adults at risk of mobility disability [46, 47] (Table 1). The intervention did not show the effects on cognitive outcomes [48]. However, in subgroup analyses amongst individuals aged ≥ 80 years and individuals with a low level of physical activity at baseline the intervention had a beneficial effect on executive functioning [48] (Table 1). A meta‐analysis on RCTs conducted amongst community‐dwelling older people suggests that physical activity serves as a preventive measure for ADL disability [49].

### Education and cognitive training

Education is a classical indicator of socio‐economic status, and individuals with lower education are at a greater risk of developing dementia and disability compared to individuals with a higher education [50, 51]. Those with low levels of education also manifest cognitive symptoms with fewer pathological changes present than individuals with high levels of education [52]. This has been suggested to be due to greater cognitive reserve amongst those with higher education. Cognitive reserve refers to the ability of the brain to cope with or compensate for neuropathology or damage. Cognitive reserve has been proposed to reduce the risk of clinical onset of dementia and cognitive decline [3, 53]. Furthermore, increasing cognitive activity has been shown to have a buffering effect against rapid cognitive decline [54].

Cognitive training and cognitive stimulation therapy have been studied both amongst healthy individuals and amongst individuals with cognitive impairment. Findings on cognitive training have shown beneficial effects, especially on the targeted cognitive functions [55, 56, 57, 58, 59] and on ADL disability [55, 58, 60, 61] in both populations. In the ACTIVE study, the effects of different types of cognitive training (memory, reasoning, speed of processing) were studied amongst volunteered older adults [62] (Table 1). Each intervention, including training of 5–6 weeks and booster sessions at 11 and 35 months for a subsample, improved the targeted cognitive abilities compared with baseline. The intervention showed no effects on daily functioning at 2 years of follow‐up [62]. However, at 5 years reasoning group reported less difficulty in IADL than the control group [61], and at 10 years of follow‐up, each intervention group reported less difficulty with IADLs [60]. Overall, the quality of the evidence on the effects of cognitive training is low [4]. Cognitive training studies are subjected to a range of limitations, and it is not yet clear whether cognitive training can reduce cognitive decline and disability. Regardless of these uncertainties, cognitive training may be offered to older adults with normal cognition and with mild cognitive impairment to reduce the risk of cognitive decline and/or dementia [4].

### Psychosocial factors and social activity

Social engagement is an important predictor of well‐being throughout life. Evidence from observational studies and nonrandomized interventions indicates that social engagement may reduce the risk of developing both dementia and physical disability through behavioural, psychosocial and cognition‐related pathways [63, 64, 65, 66]. Higher social engagement alone [67] and in combination with light physical activity and cognitive activities [68] may reduce the risk of cognitive decline and dementia. Altogether, low social participation, low number of social contacts and loneliness have been associated with cognitive decline and higher rates of incident dementia [69, 70]. Evidence from RCTs has shown positive effects of social interaction and psychosocial interventions on cognitive abilities [71, 72, 73]. However, in all, evidence from RCTs is insufficient to demonstrate the efficacy of social activity with risk of cognitive decline [4].

Stress is a risk factor for dementia and cognitive decline, and even mild‐to‐moderate psychological distress and stress have been shown to have a considerable impact on the incidence of dementia and disability in observational studies [74, 75, 76]. When it comes to depression, a substantial body of evidence links depression to cognitive decline and dementia [77] and to disability [78]. However, there is currently insufficient evidence to recommend the use of antidepressant medication for reducing the risk of cognitive decline and/or dementia [4]. Alongside these negative aspects of psychosocial functioning, further studies should look more into measures reflecting the positive aspects of psychosocial functioning, such as the happiness [79], and investigate how they predict physical and cognitive decline.

### Vascular risk factors and weight management

Vascular risk factors, such as midlife obesity, high total cholesterol level and high systolic blood pressure, are tightly associated with both dementia and disability risk later in life [80, 81], and findings related to dementia suggest that clustering of vascular risk factors increases the risk additively [80]. Moreover, vascular risk factors have been shown to be associated with structural brain changes over the life course starting from young adulthood [82]. Treating vascular risk factors with antihypertensives and statins is associated with reduced dementia risk in observational studies [83, 84], and there is some evidence from RCTs, suggesting beneficial effects of treating hypertension. The SPRINT Memory and Cognition IN Decreased Hypertension (SPRINT MIND) trial suggested that the intensive blood pressure control compared with standard treatment may prevent cognitive impairment [85] and further trials are needed to confirm these findings.

With regard to obesity, based on a recent meta‐analysis on observational studies with approximately 600 000 individuals, it was shown that obesity, but not overweight, in midlife increases the risk of dementia in later life by 33% [86]. Observational studies also demonstrate the association of obesity with an increased risk of incident disability [87]. However, evidence from weight management interventions on reducing the risk of cognitive decline and dementia is considered only low to moderate [4]. Regarding interventions with disability outcomes, recent findings amongst obese older adults suggest that caloric restriction combined with aerobic exercise training is more effective in reducing disability as compared to aerobic exercise alone [88].

### Morbidity and healthcare interventions

The role of noncommunicable diseases (NCDs) is important in the development of dementia and disability [35, 81]. For example, diabetes, heart diseases, pulmonary diseases, nonalcoholic fatty liver disease and chronic kidney disease have been linked with dementia and disability risk [89, 90, 91, 92]. Diabetes increases the risk of all‐cause dementia, AD and vascular dementia. The risk of dementia is increased by approximately 60% amongst persons with diabetes [93]. So far, there is not enough evidence to support that the intensive treatment of diabetes would be beneficial for cognition. Multimorbidity is common in older age groups, and it is a well‐known risk factor for disability [35]. Emerging evidence from recent studies suggests that multimorbidity has an important role also regarding dementia and cognitive decline [35].

Comprehensive geriatric assessment is the gold standard care for frail older people in hospital [94]. Comprehensive geriatric assessment is linked to reduced disability and greater likelihood of returning home after hospital admission [94, 95]. Also, other types of healthcare interventions have been conducted (Table 1), but the effects of these trials on disability have been very small [96, 97]. In‐home visits including multidimensional geriatric assessment and quarterly follow‐up have shown to be effective in terms of ADL disability amongst individuals with low baseline risk of nursing home admission [98] (Table 1). However, no intervention effects on cognitive functioning were found during the three‐year follow‐up [98]. A systematic review on the effects of preventive home visits suggests that some interventions might offer some cost‐neutral positive effects on physical functioning, quality of life and/or mortality [99].

### Nutrition

Healthy diet throughout life plays a central role in maintaining health and preventing NCDs. Both single nutrients and foods and dietary patterns have been investigated in relation to dementia and disability. The Mediterranean diet, a diet rich with unsaturated fats and antioxidants, is the most extensively studied dietary approach. Also, other healthy dietary patterns exist, and common features of the different patterns include high consumption of fruits and vegetables, unsaturated fats, fish and whole‐grain cereal products. Systematic reviews of observational studies have concluded that high adherence, but not modest adherence, to the Mediterranean diet is associated with a reduced risk of mild cognitive impairment and AD [100, 101]. High adherence to the Mediterranean diet has been shown to decrease the risk of incident basic ADL and IADL disability [102] and risk of incident mobility disability [103]. Nordic diet (higher intake of Nordic fruits, vegetables, cereals, low‐fat milk and fish, and lower intake of red meat, total fat and alcohol) has also been shown to protect from mobility limitations and difficulties in basic ADLs [104].

In the Older People And n–3 Long‐chain polyunsaturated fatty acids (OPAL) Study, the effects of a 24‐month dietary intervention including daily supplementation of omega‐3 fatty acids (polyunsaturated fatty acids, PUFAs) on cognition were studied amongst older cognitively healthy adults [105] (Table 1). No significant effect of the intervention as compared to olive oil placebo was found. Several systematic reviews and meta‐analyses of RCTs concerning the effects of nutrition on the risk of cognitive decline and dementia have been carried out. These include meta‐analyses concerning PUFAs [106], vitamin B [106], vitamin E [106], polyphenols [107], supplements of multi‐complexes [108, 109], protein supplementation [107] and the Mediterranean diet [110]. These studies provide moderate evidence on the beneficial effects of the Mediterranean diet in reducing the risk of cognitive decline and dementia [4], but not evidence to support the use of dietary supplements. Much less data are available on disability outcomes and nutrition. Regarding physical functioning in general, evidence from interventions points out the importance of sufficient protein gain [111]. Findings from observational studies indicate that low serum concentrations of vitamins B6 and B12 and selenium predict ADL disability [112], whereas low serum vitamin D has been shown to predict mobility disability [113]

### Smoking and alcohol

Smoking is a major risk factor for NCDs and premature death, and is also one of the leading risk factors for disability [114]. Evidence regarding dementia shows that former/active smoking is also related to a significantly increased risk of AD [115]. Combinations of nonpharmacological, including different behavioural/psychological strategies, and pharmacological approaches appear to be the most effective way in supporting smoking cessation [116]. However, interventions for smoking cessation offer only low evidence for reducing the risk of cognitive decline and dementia [4]. Nevertheless, other health benefits of smoking cessation are undisputable.

Excessive alcohol use is also one of the leading risk factors associated with death and disability [117]. Based on observational evidence, interventions aimed at reducing or ceasing harmful alcohol use should be offered to adults with normal cognition and mild cognitive impairment to reduce the risk of cognitive decline and/or dementia [4].

### Other factors

Hearing, visual and olfactory impairment may increase the risk of cognitive impairment and disability [118, 119]. Of the sensory impairments, the effects of hearing impairments [120] are studied the most. Based on a meta‐analysis of 4 prospective studies, individuals with hearing impairment had an increased risk to develop cognitive impairment compared to those without impairment [121]. In cross‐sectional studies, associations between hearing impairment and lower physical functioning, indicative of higher risk of disability, have also been reported [122, 123]. However, the evidence for possible benefits of a hearing aid is insufficient, and the use of hearing aids to reduce the risk of cognitive decline and/or dementia is not recommended [4]. Furthermore, it is not clear whether sensory impairments have a causal role in the biological processes leading to dementia and disability or whether sensory impairments follow pathological processes that have causal links to dementia and disability.

More novel possible risk factors that have been associated with dementia and disability include air pollution [124], poor sleep [125], poor dental hygiene [126, 127] and imbalance in the microbiota of the gut [127, 128]. Also, traumatic brain injuries have been linked to dementia and disability [129].

## Multidomain approach to prevent cognitive impairment and disability: RCT evidence

Late‐life cognitive impairment and dementia are complex disorders with multifactorial aetiologies and single‐domain interventions (focusing on one risk factor at time) may have only limited prevention potential. Recently, multidomain interventions targeting several lifestyle‐related factors simultaneously have gained increasing interest. In Table 2, we have gathered evidence from large (sample size of at least 500 participants) multidomain RCTs with cognitive decline or dementia or disability as the primary outcome.

**Table 2:** Completed large (over 500 participants) multidomain randomized controlled trials to prevent cognitive impairment and/or incident disability amongst older adults

Study, country	Intervention; duration	Number of participants and inclusion criteria; recruitment strategy	Outcome measures on cognition and disability	Primary outcome results	Secondary cognitive outcome results; other results/conclusions on cognitive outcomes	Secondary disability outcome results; other results/conclusions on disability outcomes
FINGER, Finland [130, 133]	Multidomain intervention including nutritional guidance, physical exercise, cognitive training, social activity and management of metabolic and vascular risk factors versus general health advice (control); 2‐year intervention	1260 participants aged 60–77 years who had an elevated risk of dementia based on CAIDE risk score ≥ 6 points, and cognitive function at or slightly below average level; participants from previous population‐based national surveys; individual randomization	*Primary outcome:* cognitive performance measured with NTB (a composite measure of 14 standard cognitive tasks) *Secondary outcomes:* NTB domain Z scores for executive functioning, processing speed and memory; ADL disability and short physical performance battery	Intervention had beneficial effect on NTB: between‐group intervention versus control difference for NTB change 0.022 (*P *= 0.030) per year	Beneficial effect of intervention on executive functioning (*P *= 0.039) and processing speed (*P *= 0.029) but not on memory (*P *= 0.36); beneficial effect of intervention on memory when including more complex memory tasks (*P *= 0.036) and higher risk of decline in cognition in the control group than in the intervention group	ADL disability score slightly increased in the control group but remained relatively stable in the intervention group (change between intervention and control − 0.95, 95% CI: −1.61 to − 0.28, after 1 year and − 1.20, 95% CI: −2.02 to − 0.38, after 2 years; in chair rise, the intervention group had a slightly higher probability of improvement (from score 3 to score 4; *P *= 0.041) and a lower probability of decline (from score 3 to scores 0–2; *P *= 0.043) compared with the control group.
MAPT, France [134]	Omega‐3 PUFA supplementation alone or in combination with multidomain intervention (cognitive training, physical activity and nutritional advice), compared with placebo capsule alone or in combination with the multidomain intervention; 3‐year intervention	1680 participants aged ≥ 70 years with memory complaint, IADL limitation or slow gait speed; recruitment using diverse strategies including patient databases and advertisements; individual randomization	*Primary outcome:* Cognition measured with composite Z score combining four cognitive tests *Secondary outcomes:* individual components of the composite score, scores on other cognitive tests (MMSE score, Trail Making Tests A and B, Controlled Oral Word Association Test and visual analogue scales; ADL disability (Alzheimer’s Disease Cooperative Study ADL Prevention Instrument), short physical performance battery, Fried’s frailty	No significant difference in cognition between any of the three intervention groups compared with placebo: Between‐group difference was 0.093 (*P *= 0.142) for multidomain + PUFA, 0.079 (*P *= 0.179) for multidomain and 0.011 (*P *= 0.812) for PUFA compared with placebo	Multidomain + PUFA had less decline (*P *= 0.036) in ten MMSE orientation items than the placebo group; other group comparisons and other cognitive outcomes showed no effect; less cognitive decline in those who received multidomain intervention (two groups pooled) than in those who did not (other two groups pooled) (*P *= 0.015); beneficial effect of multidomain plus PUFA versus placebo amongst those with CAIDE score ≥ 6; beneficial effect of multidomain + PUFA (*P *< 0.001) and multidomain (*P *= 0.003) groups versus placebo amongst those with amyloid positivity	No significant difference in ADL disability, short physical performance battery or Fried’s frailty between any of the three intervention groups compared with placebo
PreDIVA, the Netherlands [135]	Multidomain cardiovascular intervention (advice) versus usual care (control); 6‐year intervention	3526 community‐dwelling participants aged 70–78 years; recruited from general practices; cluster randomization of 116 general practices	*Primary outcome:* cumulative incidence of dementia and disability score (ALDS) at 6 years of follow‐up *Secondary outcomes:* cognitive decline as measured by MMSE and VAT; dementia subtype	No effect of intervention on mean dementia and disability scores (adjusted mean difference: –0∙02, 95% CI: –0∙38 to 0∙42; *P *= 0.93)	No effect of intervention on dementia incidence, MMSE and VAT, no effect of intervention on AD; reduced risk of non‐AD dementia in the intervention group (*P *= 0.007); reduced risk of dementia in participants with untreated hypertension at baseline who were adherent to the intervention (*P *= 0.02)	
GeMS, Finland [137]	A comprehensive geriatric assessment with a multifactorial intervention including individualized referrals, recommendations, physical activity counselling and supervised resistance training versus control (no contact); 2‐year intervention; 1‐year follow‐up	781 participants aged 75–98 years; population‐based sample of persons aged ≥ 75 years living in the area of Kuopio, Finland; random assignment to intervention and control group (no contact)	*Primary outcome:* mobility disability (self‐reported inability to walk 400 m independently)	Intervention had beneficial effect on mobility; intervention versus control: OR for mobility disability 0.82 (95% CI: 0.70–0.96) at the end of intervention and 0.84 (95% CI: 0.75–0.94) at 1 year postintervention		The positive effect of the intervention on mobility was even greater amongst persons with musculoskeletal pain

ALDS, Academic Medical Center Linear Disability Score; CAIDE, Cardiovascular Risk Factors, Aging and Dementia; FINGER, Finnish Geriatric Intervention Study to Prevent Cognitive Impairment; GeMS, Geriatric Multidisciplinary Strategy for the Good Care of the Elderly; HR, hazard ratio; IADL, instrumental activities of daily living; MAPT, The French Multidomain Alzheimer Preventive Trial; MMSE, Mini‐Mental State Examination NTB, neuropsychological test battery; OR, odds ratio; PreDIVA, The Prevention of Dementia by Intensive Vascular Care; PUFAs, polyunsaturated fatty acids; VAT, Visual Attention Test.

The Finnish Geriatric Intervention Study to Prevent Cognitive Impairment (FINGER) was the first large multidomain trial to demonstrate that it is possible to maintain cognitive functions and prevent cognitive decline with the multidomain approach amongst at‐risk older persons with existing dementia‐related risk factors [130]. In the FINGER trial, the 2‐year intervention comprised of nutritional guidance, physical exercise, cognitive training, social activity and management of metabolic and vascular risk factors, whereas the control group received general health advice. After two years, the intervention showed significant beneficial effects on global cognition (measured with neurological test battery, NTB; 25% higher improvement in the intervention group), executive functioning (83% higher improvement in the intervention group) and processing speed (150% higher improvement in the intervention group) and the risk of cognitive decline was significantly higher (30%) in the control group [130]. FINGER multidomain intervention had significant benefits also on other health‐related outcomes, including body mass index, dietary habits, physical activity [130], health‐related quality of life [131] and development of new chronic diseases [132] and in preventing ADL disability [133].

Two other large multidomain lifestyle‐based prevention trials have also been recently completed: the French Multidomain Alzheimer Preventive Trial (MAPT) [134], which tested the association of a lifestyle intervention with omega‐3 fatty acid supplements, and the Dutch Prevention of Dementia by Intensive Vascular Care (PreDIVA) [135], mainly focused on the pharmacological management of vascular/metabolic risk factors. Both trials reported no benefits of the intervention on the primary outcome, but subgroup analyses suggested cognitive benefits in subpopulations of participants with increased risk of dementia [134, 135].

Other reasons for the success of the FINGER intervention, in addition to the multidomain approach, were most likely the criteria through which study participants were chosen for the study. The CAIDE dementia risk score [136] was used to select participants who had modifiable risk factors for cognitive decline. In other multimodal RCTs published after the FINGER, participants included general community‐dwelling populations or persons with subjective memory complaints [134, 135], and in the primary analyses, the benefits of the intervention have not become evident. However, when the effects of the intervention have been investigated amongst individuals with risk factors for dementia (elevated CAIDE score or untreated hypertension), a positive effect was observed.

One large Finnish trial, Geriatric Multidisciplinary Strategy for the Good Care of the Elderly (GeMS), has examined the effects of comprehensive geriatric assessment in combination with individually tailored intervention on mobility disability [137]. The two‐year intervention, which included also supervised resistance training, had beneficial effects on mobility, thus preventing mobility disability. Subgroup analyses showed that the positive effect on mobility was even greater amongst persons with musculoskeletal pain [137]. In all, evidence from a large meta‐analysis on RCTs including nearly 100 000 individuals shows that multidomain interventions can improve physical functioning and independence in older adults [138]. These results support the conclusion that multidomain approaches targeting several lifestyle risk factors simultaneously are most likely an effective way to comprehensively support healthy ageing.

Especially regarding dementia and cognitive decline, the significance of different risk factors may vary largely between individuals and across population groups. This most likely applies also to physical disabilities. This means that preventive measures should be more and more individually tailored. Further research is needed to establish whether specific combinations of risk factors induce greater risk than others [139]. In addition, several methodological considerations should be taken into account when planning a preventive intervention, such as timing of the intervention, choosing outcome measures that are sensitive enough to detect early changes and doing the right things and enough of them [140].

## Next steps in dementia and disability prevention

### Global collaboration: World‐Wide FINGERS network

Following the positive results of the FINGER trial, several countries worldwide are now planning their own interventions following the FINGER model. To support this global work, the World‐Wide FINGERS Network (www.alz.org/wwfingers) has been launched. The aim of this global network is to test, adapt and optimize the FINGER intervention in diverse geographical and cultural settings [141, 142]. Today around 30 countries are planning or conducting their multidomain interventions to prevent dementia and disability. In addition, new technologies and eHealth solutions utilizing multidomain approach are being tested and may facilitate personalized, effective and feasible interventions and implementation [143].

### Addressing emerging health issues for older adults: the role of COVID‐19 and other infections

Older frail persons and persons with cognitive impairment are vulnerable for other types of environmental risks as the SARS‐CoV‐2 infection (COVID‐19) has demonstrated. The severe and fatal cases of SARS‐CoV‐2 infection are higher in older adults with preexisting health conditions and multimorbidity [144]. National health systems are currently forced to reduce disease management for NCDs [145]. The forced lockdown and reduced monitoring can impact the current and future health and well‐being of seniors, especially those with multiple risk factors and NCDs, through several mechanisms (e.g. biological, social). The length of the COVID‐19 emergency may be much longer than originally expected and there may be reoccurrence, increasing susceptibility for negative health outcomes also amongst uninfected individuals. Thus, it will be important to identify factors that can influence and predict the short‐ and long‐term health‐related outcomes of COVID‐19 outbreak in seniors and develop prediction and decision models to optimize the management of this and similar type of outbreaks in seniors.

### Implementation of research evidence

Large body of evidence is showing that even if not curable, a lot can be done to slow down the progression of both disability and cognitive decline. By supporting healthy lifestyle choices, social activity and providing adequate health and social care services, the burden of dementia and disability can be most likely reduced.

In 2017, the WHO launched a global action plan on the public health response to dementia 2017–2025 [146]. To support dementia risk reduction in different countries, the WHO published guidelines on risk reduction in cognitive decline and dementia [4]. These guidelines are an important tool for healthcare providers, governments, policymakers and other stakeholders to strengthen their response to the dementia challenge. The guidelines highlight that many of the modifiable risk factors for dementia are shared with other noncommunicable diseases (NCDs), and therefore, the recommendations aiming to prevent cognitive decline should be integrated into already existing programmes for diabetes and cardiovascular disease risk reduction. Since dementia together with diabetes and cardiovascular diseases are important causes for disability amongst older adults, actions aiming to prevent or postpone the onset of these noncommunicable diseases are likely to have remarkable effects on disability prevention as well.

Already now, several countries have taken concrete steps in the field. Alzheimer’s Disease International (ADI) has launched a report [147] that supports the implementation of WHO’s risk reduction guidelines and provides also an overview of actions that have been taken place in response to WHO’s global action plan on the public health response to dementia 2017–2025. However, there are still areas that need to be further developed. For example, most national plans focus on dementia awareness and support, and risk reduction is included only in the minority of the plans. In addition, less than half of national plans have received funding for effective implementation. In the future, it would be important to secure the funding to implement the national plans, to highlight more the importance of early prevention of both disability and dementia and to start effectively implementing the WHO’s risk reduction guidelines to national healthcare policies and concrete actions.

## Future perspectives

Within the next decade, as the World‐Wide FINGERS Network RCTs are being completed and data are being analysed, we can expect to gain deeper understanding on the feasibility and efficacy of nonpharmacological approaches for dementia and disability prevention for different populations and settings. The World‐Wide FINGERS Network is also working towards the development of preventive models combining nonpharmacological and pharmacological interventions.

Although disease‐modifying drugs for AD are not yet available, several compounds are being tested in RCTs, with an increasing number of agents targeting pathophysiological pathways other than amyloid and tau [148]. Particularly, innovation in drug development for neurodegeneration is brought by the increasing presence of compounds targeting biological processes driven by ageing, which are involved in onset and development of different age‐related chronic diseases causing disability. Age‐related biological processes relevant to neurodegeneration include systemic inflammation, impaired autophagy and clearance of misfolded proteins, vascular dysfunction, epigenetic dysregulation, mitochondrial and metabolic dysfunctions, and synaptic dysfunction and loss [149]. Compounds targeting these mechanisms include also agents identified through drug‐repurposing strategies, which may accelerate the identification of safe and effective treatments [148, 149].

The concept of combination therapy, which is already a standard practice for many chronic disorders (e.g. heart failure, cancer), is also gaining interest in the dementia field, as an effective way to address the heterogeneity of the majority of dementia cases in older adults. Finally, progresses in the identification of noninvasive or minimally invasive biomarkers for early detection of AD risk, including blood‐based biomarkers, will facilitate large‐scale approaches for risk assessment and early interventions [150, 151]. The large‐scale dissemination and implementation of scientific results can be supported by bodies such as the WHO, which through the global action plan on the public health response to dementia 2017–2025 can support dissemination of evidence‐based practice for dementia risk reduction, and coordinate multisectoral collaboration for public health prevention programmes [146].

## Summary and Conclusions

Preventive measures to tackle both dementia and disability are of utmost importance, not only for the individual, but also for the society given the substantial burden they cause. There is increasing evidence that several environmental factors throughout the life course have a significant role for the risk of cognitive impairment and dementia. The most established modifiable risk factors are physical inactivity, cardiovascular diseases, diabetes mellitus, hypertension, obesity, depression and smoking. Especially, by targeting several modifiable risk factors at a time can prevent or postpone dementia and disability. Close collaboration with researchers, policymakers, healthcare practitioners, civil society, at‐risk persons and persons who live with dementia and disabilities is the way towards healthier and age‐friendly ageing societies.

In Table 3, we have collected key points of the review.

**Table 3:** Key points and future directions for dementia and disability prevention

Key clinical points
Detection of modifiable risk factors for dementia and disability in older adults (and possibly, also in midlife adults) can help identify individuals who can benefit from preventive interventions For cognitive impairment and dementia, the level of evidence for some interventions to reduce risk factors still needs to be strengthened. However, interventions addressing these risk factors are still relevant for other health benefits A person‐centred approach, adequate information and engagement of the individual can increase awareness of the at‐risk status and ameliorate adherence to preventive measures
Recommendations for future research
Ongoing, large‐scale RCTs are evaluating the feasibility and efficacy of multidomain interventions in delaying or preventing cognitive impairment, dementia and disability. If positive effects will be confirmed, public health strategies for a life‐course‐based implementation of these interventions in the community needs to be developed Optimization of the efficacy and the long‐term sustainability of these preventive interventions will require precision‐based/personalized approaches and will be facilitated by eHealth, mHealth and ICT Tools for risk assessment, intervention delivery and monitoring
Additional resources for healthcare professionals
WHO guidelines for Risk Reduction of Cognitive Decline and Dementia: https://www.who.int/mental_health/neurology/dementia/guidelines_risk_reduction/en/ WHO International Classification of Functioning, Disability and Health (ICF): https://www.who.int/classifications/icf/en/

## Author contribution

**Inna Lisko:** Conceptualization (supporting); Funding acquisition (equal); Project administration (supporting); Visualization (lead); Writing‐original draft (lead); Writing‐review & editing (equal). **Jenni Kulmala:** Conceptualization (supporting); Funding acquisition (equal); Project administration (supporting); Supervision (supporting); Writing‐original draft (supporting); Writing‐review & editing (equal). **Martin Annetorp:** Funding acquisition (equal); Writing‐review & editing (equal). **Tiia Ngandu:** Conceptualization (supporting); Funding acquisition (equal); Project administration (supporting); Writing‐review & editing (equal). **Francesca Mangialasche:** Conceptualization (supporting); Funding acquisition (equal); Visualization (lead); Writing‐original draft (supporting); Writing‐review & editing (equal). **Miia Kivipelto:** Conceptualization (lead); Funding acquisition (equal); Project administration (lead); Resources (lead); Supervision (lead); Visualization (supporting); Writing‐review & editing (equal).

## Conflict of Interest

The authors declare no conflict of interest.
